# The First-Line Approach in Children with Obstructive Sleep Apnea Syndrome (OSA)

**DOI:** 10.3390/jcm12227092

**Published:** 2023-11-14

**Authors:** Nicole Mussi, Roberta Forestiero, Giulia Zambelli, Letizia Rossi, Maria Rosaria Caramia, Valentina Fainardi, Susanna Esposito

**Affiliations:** Pediatric Clinic, Department of Medicine and Surgery, University of Parma, 43126 Parma, Italy; nicole.mussi1@gmail.com (N.M.); forestiero.roberta@gmail.com (R.F.); zambelligi@gmail.com (G.Z.); letiziarossi95@libero.it (L.R.); maracaramia91@gmail.com (M.R.C.); valentina.fainardi@unipr.it (V.F.)

**Keywords:** adenotonsillar hypertrophy, intranasal steroids, leukotriene receptor antagonists, obstructive sleep apnea syndrome, polysomnography, sleep disordered breathing

## Abstract

Obstructive sleep apnea syndrome (OSA) is the main manifestation of sleep-disordered breathing in children. Untreated OSA can lead to a variety of complications and adverse consequences mainly due to intermittent hypoxemia. The pathogenesis of OSA is multifactorial. In children aged 2 years or older, adenoid and/or tonsil hypertrophy are the most common causes of upper airway lumen reduction; obesity becomes a major risk factor in older children and adolescents since the presence of fat in the pharyngeal soft tissue reduces the caliber of the lumen. Treatment includes surgical and non-surgical options. This narrative review summarizes the evidence available on the first-line approach in children with OSA, including clinical indications for medical therapy, its effectiveness, and possible adverse effects. Literature analysis showed that AT is the first-line treatment in most patients with adenotonsillar hypertrophy associated with OSA but medical therapy in children over 2 years old with mild OSA is a valid option. In mild OSA, a 1- to 6-month trial with intranasal steroids (INS) alone or in combination with montelukast with an appropriate follow-up can be considered. Further studies are needed to develop an algorithm that permits the selection of children with OSA who would benefit from alternatives to surgery, to define the optimal bridge therapy before surgery, to evaluate the long-term effects of INS +/− montelukast, and to compare the impact of standardized approaches for weight loss.

## 1. Introduction

Sleep-disordered breathing (SDB) refers to an upper airway dysfunction during sleep characterized by snoring and/or increased respiratory effort secondary to increased upper airway resistance and pharyngeal collapsibility [1,2,3]. Obstructive sleep apnea syndrome (OSA) is the main manifestation of SDB in children [4]. OSA is characterized by partial and prolonged obstruction (hypoventilation) and/or intermittent partial (hypopnea) or complete (apnea) obstruction of the upper airways. This causes an absence of airflow despite continuous respiratory effort and is usually associated with reduced peripheral oxygen saturation and/or hypercapnia [5]. In children, OSA prevalence is between 2% and 5.7%, and it is more common in males and in preschool age [1,2,3,4,6].

OSA symptoms are classically divided into diurnal and nocturnal. During the daytime, nasal speech, restless sleep, inattention, morning headaches, mood instability, depression, irritability, aggressiveness, and learning difficulties are reported [5,7]. On the other side, nocturnal symptoms include snoring, witnessed apnea, oral breathing, paradoxical thoracic movements, abnormal sleeping positions, frequent awakenings, intense sweating, nightmares, sleepwalking, nocturnal enuresis, and diaphoresis [5,7]. Untreated OSA can lead to a variety of complications and adverse consequences, mainly due to intermittent hypoxemia [5]. Children with OSA have an increased risk of cardiovascular and pulmonary complications, such as elevated blood pressure, pulmonary hypertension, or right heart failure; they may present growth failure or develop early metabolic syndrome [7] and, due to the fragmentation of the normal sleep pattern, they may present cognitive or neuropsychological function deficits (i.e., deficit in general or verbal intelligence, executive functions, learning, memory, visuospatial skills, language, mathematical skills, and abstract and analytical thinking) [5,8].

The diagnosis of pediatric OSA can be determined objectively by polysomnography (PSG) or, if polysomnography is not available, by nocturnal pulse oximetry [1,2,3,4,5]. Furthermore, a drug-induced sleep endoscopy (DISE) is a diagnostic tool that could be used to assess the upper airway of snorers and OSA patients in conditions that mimic natural sleep [4]. In addition, a detailed clinical history including nocturnal and diurnal symptoms and specific clinical and anamnestic questionnaires like the Pediatric Sleep Questionnaire [9] or the Teenager STOP-BANG questionnaire [10] can contribute to the diagnosis. During clinical examination, evidence of tonsillar hypertrophy, adenoid facies, dental malocclusion, craniofacial malformations, and neuromuscular or neurological disease should be evaluated because they can be associated with OSA [4]. Based on an objective examination, OSA has three phenotypes [10]: classic phenotype, characterized by adenotonsillar hypertrophy with or without dental and skeletal malocclusion; adult phenotype, characterized by obesity associated or not with aspects of the classical phenotype; congenital phenotype, characterized by craniofacial anomalies associated with genetic syndromes (i.e., Pierre Robin sequence, Crouzon syndrome, Apert syndrome, and Down syndrome).

The pathogenesis of OSA Is multifactorial. In children aged <23 months, craniofacial abnormalities such as alterations of size, position, and geometry of the mandible and tongue play an important role in the obstruction of the upper airways (as in the Pierre Robin sequence) [1,5]. Some clinical syndromes such as Down, Prader–Willi, Beckwith–Wideman, or Noonan syndrome, achondroplasia, Ehlers–Danlos syndrome, sickle cell disease, or any conditions that involve the respiratory control center, are strongly associated with OSA [5,6]. Other causes include laryngeal or nasal obstructions and neurological and neuromuscular diseases [5]. In children aged 2 years or older, adenoid and/or tonsil hypertrophy are the most common causes of upper airway lumen reduction; obesity becomes a major risk factor in older children and adolescents since the presence of fat in the pharyngeal soft tissue reduces the caliber of the lumen [2,3,5]. Allergic rhinitis due to nasal mucosa inflammation can increase airway resistance and favor OSA [7,11].

Experts recommend a multidisciplinary approach in the diagnosis, treatment, and care of children with OSA and two different pathways, depending on the age of the patient (< or >2 years) [1,2,3,4,5]. In particular, the treatment of OSA depends on severity, etiology, and comorbidities. A multidisciplinary team that includes a family pediatrician, pulmonologist, otorhinolaryngologist, child neuropsychiatrist, orthodontist, and other specialists (i.e., maxillofacial surgeon, cardiologist, anesthetist) shares the management of the child with OSA. Treatment options include surgical and non-surgical options. Surgical options are represented by adenotonsillectomy (AT), the gold standard for adenoid hypertrophy, and surgical corrections of craniofacial anomalies in younger children. Non-surgical treatment includes medications, orthodontic and myofascial treatment, and ventilatory therapy. However, there are still some controversies in the non-surgical approach to OSA. This narrative review summarizes the evidence available on the first-line approach in children with OSA, including clinical indications for medical therapy, its effectiveness, and possible adverse effects. The MEDLINE–PubMed database was searched to collect and select publications from 2003 to 2023. The following combinations of keywords were used: “obstructive sleep apnoea syndrome” OR “OSA” OR “OSAS” OR “sleep disordered breathing” AND “children” OR “pediatric” OR “pediatric” OR “adolescent”. We also performed the manual search of the reference lists of the selected studies. The search was limited to English-language journals and full papers only, as well as manuscripts that reported a medical approach.

## 2. Adenotonsillar Hypertrophy

Adenoids are masses of lymphoid tissue located in the posterior wall and roof of the nasopharynx covered by a pseudostratified columnar ciliated epithelium bent forming surface folds; whereas tonsils are masses of lymphoid tissue placed in the lateral wall of the oropharynx covered by squamous stratified non-keratinizing epithelium [12]. Adenoids have an immunity role, representing the first lymph node stations of the respiratory and digestive tract and belonging to the Waldeyer ring, which has a defensive action against microorganisms. Here, B and T cells are produced and also immunoglobulins. The volume of tonsils depends on age, hereditary factors, and pathological conditions; tonsils increase in size until 5 or 6 years of age and then reach their maximum volume around puberty, measuring approximately 20–25 × 10–15 mm [13]. Instead, adenoids increase in size with age, until five or six years of age, and then gradually decrease by the age of eight or nine years [13]. 

About the etiology, infectious stimuli and external aggravations may cause adenotonsillar hypertrophy [11]. In the first group, microorganisms may generate an increase in lymph cells in the tonsils and adenoids, as confirmed by the greater presence of pathogenic bacteria, in particular *Haemophilus influenzae*, *Streptococcus pneumoniae*, and other B-lactamase-producing microorganisms found in the culture of patients with hypertrophic tonsils and adenoids, compared to non-hypertrophic tonsils and adenoids [13,14,15]. In the second group, external aggravations, for example, smoke and tobacco, can determine an alteration of the microbial environment in the lymphoid tissue [15].

While adenoid hypertrophy is clearly associated with a risk of OSA, there is no consensus on the association between tonsillar hypertrophy and OSA. However, when adenotonsillar hypertrophy is suspected, a good anamnesis must be collected to assess mouth breathing, snoring, dysphagia, headaches, dyspnea, frequent awakenings during the night, somnolence during the day, enuresis, difficulty concentrating, poor school performance, sialorrhoea during sleep, and rhinolalia [13]. Upon physical examination, we could find the typical “adenoid facies”, characterized by a long, lean face with an open mouth, arched palate, underdeveloped upper jaw bones (i.e., hypoplasic maxilla), short upper lip, elevated nostrils, and dental crowding of the front teeth [13]. Instrumental examinations useful for diagnosis include lateral neck X-ray, videofluoroscopy, and nasal endoscopy; nasal endoscopy performed by the otolaryngologist represents the gold standard [16,17]. Tonsillar hypertrophy is measured with Brodsky’s grading scale [18]: grade 0, tonsils confined in tonsillar fossa; grade 1, oropharyngeal width occupied less than 25%; grade 2, occupied between 26 and 50%; grade 3, occupied between 51 and 75%; and grade 4, occupied more than 75% [18,19]. Likert’s classification is used for adenoid hypertrophy [20]. It is based on the occlusion of posterior nasal aperture: grade 1, occlusion until 25%; grade 2, between 25 and 50%; grade 3, between 50 and 75%; and grade 4, occluded more than 75% [18]. Enlarged adenotonsillar tissue can occur with a wide range of symptoms: voice changes, loss of appetite, runny nose, frequent ear infections, chronic sinusitis, snoring, and sleep apnea. Sleep apnea is due to an interference with breathing during sleep caused by a blocked airway [13]. Previous studies failed to demonstrate a correlation between the severity of tonsillar hypertrophy and the severity of OSA [21,22,23,24]. However, tonsil size is slightly related to AHI, and thus with the severity of OSA [25,26]. Tonsil hypertrophy of 25–50% may lead to a two-fold increase in OSA development, hypertrophy of 50–75% to a three-fold increase, and hypertrophy of 75–100% up to an eight-fold increase [19]. An adenoidectomy and tonsillectomy are the first-line treatment for AH. AT is associated with significant improvements in sleep-disordered breathing in most children, as it is effective in 70% to 100% of patients [27]; polysomnographic findings return to normal values in 79% of patients that undergo surgery [28]. However, it seems that a “watch and wait” strategy can be equally effective. After a 7-month follow-up in children with mild–moderate OSA who had undergone early surgery or close observation with no intervention, the AHI score and neurocognitive outcomes improved in both cases [29]. AT is not curative in all cases since between 40 and 75% of children have persistent disease after surgery. Children older than 7 years, those with asthma, obesity, Down syndrome, or with severe OSA are at increased risk for residual apneas [3,30,31]. 

Many techniques have been developed for tonsillectomy over the years in an attempt to optimize efficacy and achieve minimal morbidity. Recently, the use of intracapsular tonsillectomy over extracapsular tonsillectomy to decrease the incidence of hemorrhage and postoperative recovery time was a focus of research in pediatric otolaryngology [32]. There is still limited consensus in practice on which technique provides the best long-term outcomes in pediatric patients. While experts acknowledged the promising results seen in recent research supporting the benefits of intracapsular tonsillectomy, extracapsular tonsillectomy remains the more well-studied and tested method. Clinicians remain worried about the potential for tonsillar tissue regrowth and the need for subsequent reoperation if all tonsillar tissue is not removed [32].

Otherwise, healthy children aged between 2 and 12 years with mild OSA can benefit from a period of observation and also from the medical therapy reported below if surgical intervention is not necessary. In the case of moderate OSA or mild OSA associated with poor quality of life or in children with syndromes, early AT is the preferred approach [29].

## 3. Indications to Medical Therapy in Children

In children aged >2 years, the most frequent cause of OSA is adenotonsillar hypertrophy and medical therapy can be proposed in the same cases as an alternative to AT or after surgery when AT is not successful [10]. Since AT can be associated with a certain number of complications such as nausea and vomiting, dehydration, delayed feeding, throat pain, and the more serious post-surgical hemorrhage [33], medical therapy is usually preferred in mild cases of adenotonsillar hypertrophy.

Medical therapy of adenotonsillar hypertrophy should be considered in these conditions [4,7]: when surgery cannot be performed; for children with mild and uncomplicated forms; as a bridge therapy before surgery; during Continuous Positive Airway Pressure (CPAP) application; and for residual forms after surgery.

### 3.1. Mild and Uncomplicated Forms

According to the statements provided by the European Respiratory Society, the polysomnographic classification of OSA is based on the number of apnea–hypopnea per hour (Apnea Hypopnea Index, AHI) [1,4]. Mild forms are defined for an AHI value between one and five events/hour with SpO2 nadir between 86% and 91%. These forms are also the least related to the complications of the syndrome (cardiovascular and pulmonary complications). Given the lower effectiveness of the surgery treatment in patients with mild forms and the few side effects described by these medications, medical management should be considered.

### 3.2. When Surgery Cannot Be Performed

AT is the first-line treatment in children over 2 years of age with OSA caused by adenotonsillar hypertrophy. Patients with an AHI >5 respond better to AT [4,34,35,36]. However, the surgical procedure can sometimes be associated with risks and complications, such as nausea and vomiting, dehydration, delayed feeding, throat pain, and the more serious post-surgical hemorrhage. Moreover, the risk of post-surgical respiratory complications is higher if the surgical indication is OSA [37]. Risk factors for complications are severe forms, age being <3 years old, cardiac complications, failure to thrive, upper airway infections, obesity, and also genetic, craniofacial, and neuromuscular abnormalities [7,34]. These are not contraindications to performing surgery but, if present, the patient should be admitted to the hospital for at least one night [3,30,34,36]. Therefore, AT may not be universally suitable for all children [33]. In moderate-to-severe OSA, if AT is not recommended due to the risk of complications, continuous positive airway pressure (CPAP) can be considered.

### 3.3. Bridge Therapy before Surgery

Evangelisti et al. in 2019, in an unblinded open label study, evaluated the effectiveness of systemic corticosteroids in 12 children with severe OSA and adenotonsillar hypertrophy before surgery [37]. The results suggested that systemic betamethasone (0.1 mg/kg per day for 5 days), associated with intranasal steroids, was useful as a rescue treatment. Discordant results were reported by Al-Ghamdi et al., who evaluated if a 5-day course of oral prednisone was useful in reducing symptom severity, adenotonsillar size, and polysomnographic index in children aged from 1 to 12 years with OSA [38]. They revealed that systemic steroids did not reduce the symptoms associated with OSA nor the dimensions of the tonsils and adenoids and did not result in improvements in polysomnography [38]. However, a trial with a short course of systemic steroid therapy may be attempted as bridge therapy before surgical treatment.

### 3.4. Continuous Positive Airway Pressure (CPAP)

In the case of CPAP therapy, there is not a need to provide specific medical treatment before surgery. However, intranasal steroids (INS) are commonly administered to decrease nasal congestion and rhinorrhea, the most common side effects of CPAP. They are, therefore, useful in patients with low CPAP adherence due to rhinitis [39] and in adult patients they seem to increase compliance to ventilation after 90 days of treatment [40].

### 3.5. Residual Forms after Surgery

In a large follow-up study, only 27% of children had a complete resolution of OSA after AT [27]. Risk factors for persistent OSA after AT are: age >7 years, asthma, nocturnal enuresis, allergic rhinitis, and the severity of OSA before surgery. The three major risk factors involved in the development of residual forms are: prenatal anatomical impaired development (such as micrognathia, macroglossia, and midface hypoplasia), upper airway tissue deposition or infiltration (such as obesity, mucopolysaccharidoses), and increased airway collapsibility (due to local inflammation such as in asthma or allergic rhinitis, or to neurologic alterations such as neuromuscular disease or patients with cerebral palsy) [41].

## 4. Medications

As the enlarged adenoids and tonsils consist of hypertrophied lymphoid tissue, anti-inflammatory medications are considered the first-line treatment before surgery [10,42]. These include corticosteroids, especially INS and leukotriene receptor antagonists (LTRA; i.e., montelukast).

### 4.1. Intranasal Steroids (INS)

INS have an anti-inflammatory effect that reduces the release of inflammatory mediators and inhibits inflammatory cell recruitment to the nasal mucosa [43]. It was demonstrated that inflammatory mediators and their expression is increased in the nose and pharynx of patients with OSA [44]. In support of this, neutrophilic cells in sputum and elevated inflammatory markers (leukotrienes, prostaglandins) in exhaled breath condensate were found. Furthermore, the presence of two isoforms of glucocorticoid receptors (GRC-alpha and GCR-beta) were demonstrated in adenoid and tonsil tissues [45]. It is not known whether this receptor expression is a cause or a consequence of the disease, but it is associated with a greater response to INS therapy in children with OSA [44]. Lastly, low-grade chronic systemic inflammation was also described, as demonstrated by the increase in the serum C-reactive protein and proinflammatory cytokines [10,46]. A bidirectional relationship between atopic disorders and OSA was assumed [10]. The inflammation of the upper airway, seen in children with allergic rhinitis, may lead to increased nasal resistance, sleep disorders, and therefore, OSA [47].

INS have a higher benefit-to-risk ratio than systemic corticosteroids and may be used for longer periods since they mainly act on nasal mucosa [43,44]. Local adverse effects of INS include epistaxis, nasal irritation, dry nose, candidiasis, and pharyngitis, but these are reported to be no more frequent than with a placebo [47]. The systemic effect of INS derives from the small amount absorbed across the respiratory mucosa and the small amount swallowed. The principal side effects are suppression of the hypothalamic–adrenal axis (HPA), linear-growth suppression, and bone metabolism; the risk of these side effects are low, even if used for prolonged time [11].

There are different types of corticosteroids available for intranasal use. The main used and investigated INS are budesonide, fluticasone, and mometasone. However, newer agents (i.e., fluticasone propionato and mometasone) appear to have higher topical potency than older agents (i.e., beclomethasone and budesonide) [43].

Brouillette et al. evaluated the effect of a 6-week course of intranasal fluticasone propionate (50 µg per nostril twice daily for the first week, then once daily for 5 weeks) versus placebo in 25 children with OSA [48]. The trial showed that mixed/obstructive AHI decreased from 4.9 ± 1 events per hour in the fluticasone propionate group and increased by 2.2 ± 3.3 events per hour in the placebo group. They also showed significant improvements in the frequencies of hemoglobin desaturation and respiratory movement/arousals with no change in adenotonsillar size [48]. A randomized controlled trial conducted by Chan et al. compared intranasal mometasone furoate against placebo for 16 weeks in children with mild OSA [49]. It showed an improvement in PSG parameters: the mean AHI score decreased from 2.7 ± 0.2 to 1.7 ± 0.3 events per hour in the intervention group but increased from 2.5 ± 0.2 to 2.9 ± 0.6 events per hour in the placebo group. There was a small reduction in the oxygen desaturation index in the mometasone furoate group but no significant differences were demonstrated in adenotonsillar size and the results seemed to be more significant for the subgroup with allergic rhinitis [49]. Regarding budesonide, a 6-week course of once-daily intranasal budesonide was shown to improve symptoms, reduction in adenoid size, and decrease AHI among children with mild-to-moderate OSA syndrome with a sustained effect two months after discontinuation [50].

A Cochrane review in 2020 assessed the efficacy of anti-inflammatory medications in 240 children with objectively diagnosed OSA (AHI > 1) [51]. The review included three randomized controlled trials that investigated mometasone furoate, budesonide, and fluticasone propionate. In both trials, the AHI decreased in the intervention group compared to placebo; however, based on the pooled effect estimate from the meta-analysis, they concluded that no differences could be found in the effectiveness of INS alone, maybe due to possible short-term beneficial effects to the desaturation index and SpO2 in children. It was suggested that regular follow-up visits may be necessary to detect recurrence of OSA after successful treatment [51].

However, INS has a short-term effectiveness in mild and uncomplicated forms of OSA, as demonstrated by the improvement of symptoms and polysomnographic parameters [52]. A trial should be recommended in clinical practice. On the other hand, the long-term effect on tonsil and adenoid size is more uncertain. In 2007, a single blind randomized study conducted in 178 children with adenoid hypertrophy evaluated the effect of an 8-week treatment with intranasal flunisolide against a saline solution, showing a significant reduction of adenoid hypertrophy in 72.6% of the children versus 30.7% with placebo. More specifically, in these children, the size of the adenoids decreased from VI degree to III degree, preventing the surgical treatment [52]. This effect may be caused by an anti-inflammatory mechanism and lymphocytic action in the upper airways.

Zwierz et al. analyzed the long-term effects on adenoid size and mucus in 165 children with adenoid hypertrophy grade II and III after a 12-week treatment with intranasal mometasone furoate [53]. The study showed a non-significant change in adenoid size, 3 to 6 months after treatment. On the contrary, Berlucchi et al. conducted a two-stage prospective, placebo-controlled, randomized trial to evaluate the efficacy of intranasal mometasone furoate aqueous on the improvement of adenoid hypertrophy and chronic nasal obstruction [54]. They showed successful results after long-term maintenance therapy but patients with poor compliance symptoms relapsed after discontinuance, leading to surgical treatment. Kheirandish-Gozal et al. demonstrated that intranasal administration of budesonide (32 µg per puff per nostril to both nostrils at bedtime) in children with mild OSA for 6 weeks led to an improvement in the AHI and the respiratory arousal index (RAI), some parameters of sleep microarchitecture, and a significant reduction in the size of the adenoids compared to children who received a placebo [55]. Furthermore, the beneficial effect was maintained for 8 weeks after drug discontinuation. Regarding nasal beclomethasone, an improvement was demonstrated after 2 weeks of therapy in 45% of patients (“responders”). Among these children, a further 24-week treatment improved clinical symptoms at 52 and 100 weeks and reduced the use of surgical treatment compared to “non responders” to the first 2-week therapy [56]. In addition, in another study, an 8 week trial with mometasone furoate followed by a dose on alternate days for 16 weeks seemed to be useful for obstructive adenoids when evaluated by endoscopy performed at pre-treatment and at 24 weeks post-treatment [57]. Overall, the long-term beneficial effects probably occur when INS are used as a maintenance therapy for a long period and diminish when the therapy is not administered. To avoid those minimal side effects of INS, therapy can be discontinued when symptoms are less present, for example, in the summer period, as demonstrated by the improvement of adenoid mucus and tympanometry parameters. In children aged 6–9 years, the prolonged use of mometasone furoate for one year is linked to an increased occurrence of epistasis compared to placebo [58] and the prolonged, combined use of INS and topical decongestants may cause tachyphylaxis and rebound congestion [59]. A one-year continuous treatment with INS is not associated with growth delay [58], but a regular clinical follow-up of stature should be performed [59].

### 4.2. Leukotriene Receptor Antagonists (LTRA)

Leukotrienes (LTs) are inflammatory mediators produced by mast cells, eosinophils, basophils, monocytes, and macrophages [60]. It was shown that LT pathways contribute to proliferative and inflammatory signaling. They have an immune function, stimulating the accumulation of leukocytes, the destruction of pathogens, and the production of chemokines and cytokines from the activation of NF-kB [60]. LTs act by binding to receptors located on target cells, which may be leukocytes, epithelial cells, smooth muscle cells, or endothelial cells, interacting with one or both of their related receptor classes. Once bound to the leukotrienes, these receptors interact with G-proteins in the cytoplasm, leading to increased intracellular calcium and decreased intracellular cyclic AMP [61]. 

In children with OSA, LTs are involved in the inflammation located in the upper airways. In fact, they and their receptors are overexpressed in adenotonsillar tissue and exhaled condensates of OSA patients [62,63]. In vitro, leukotrienes increase adenotonsillar cell proliferation, while this proliferation is reduced by LT receptor antagonists as well as the concentration of inflammatory mediators, such as tumor necrosis alpha, interleukin (IL)-6, and IL-12 [62,63]. The main mechanism by which LTRA improve OSA is the reduction of upper aerodigestive tract inflammation, pharmacologically interrupting the LT pathway involved in the proliferative and inflammatory signaling pathways in the adenotonsillar tissue of children with OSA [12].

Montelukast is widely used for allergic diseases such as asthma and rhinitis that often coexist with pediatric OSA, and was considered a therapeutic option for children with mild OSA due to its anti-inflammatory activity [12,22]. Dayyat et al. evaluated three different medications (montelukast, zileuton, and the cysteinyl leukotriene receptor 1 and 2 antagonists, BAY u9773): all three molecules decreased TNF-alfa, IL-6, and IL-12 levels, with selective changes in IL-8 and no effects on IL-10; montelukast proved to be the most effective, while zileuton had a lower anti-inflammatory effect requiring a higher concentration to have the same effect on the reduction of cell proliferation in the tonsils and adenoids [64]. Montelukast is a cysteinyl LT receptor antagonist, orally bioavailable, with a short-term beneficial, clinically meaningful effect on OSA in otherwise healthy, non-obese, surgically untreated children by reducing the number of apneas, hypopneas, and arousals during sleep as well as improving SpO2 during sleep [51]. Montelukast improves AHI by 55% and reduces adenoid hypertrophy and the AHI index compared to placebo (from 9.2 ± 4.1/h to 4.2 ± 2.8/h vs. no changes in the placebo group) [2]. Montelukast should be administered as a 3-month course [65]. The reduction of adenotonsillar size after a 12-week treatment was also demonstrated by Shokouhi et al. using a nasal endoscopy and neck radiography performed at baseline and after the therapeutic course [15].

Urinary cysteinyl leukotrienes (cysLTs) can be used as predictors of the efficacy of montelukast. Several studies demonstrated the positive correlation between OSA severity and cysLT levels [64,66]. Sunkonkit et al. reported higher urinary cysLT levels in subjects who responded to montelukast therapy compared to non-responders [66]. Although there are no precise reference values, the authors found that the values of urinary cysLTs E4 (the major urinary metabolite of cysLTs) >1.457 pg/mg creatinine could have a very high specificity and sensitivity in predicting the therapeutic response to montelukast [64]. The most common adverse effects of montelukast are headaches, rashes, abdominal pain, hyperkinesia, and eczema [67]. However, recent further adverse events were reported, like allergic granulomatous angiitis and sleep and psychiatric disorders [68]. Depression, suicidal ideation, and nightmares (most frequent between the ages of 6 and 10 years) were reported several times in both adults and children [30,69].

### 4.3. Intranasal Steroid and Montelukast

Compared to single medication, the combination of INS and LTRA is significantly more effective in reducing apnea and overnight symptoms with fewer benefits in those with severe adenoid hypertrophy [39,70,71,72,73]. A retrospective study demonstrated that 62% of children with mild OSA showed normal polysomnography after INS and montelukast, particularly those non-obese and younger [39]. In the meta-analysis of Liming et al. [70], both montelukast alone and in combination with INS improved the AHI and the lowest oxygen saturation. The best efficacy of combined therapy was also demonstrated in the observational study by Da-Zhi Yang [71], which compared the 12-week use of oral montelukast, mometasone furoate nasal spray and their combined use. The highest response was shown in children who received combined therapy with respiratory symptoms such as snoring and apnea and restless sleep relieved in a shorter time than using the single medication. 

In consideration of the greater efficacy of combined therapy with montelukast and INS, this therapeutic combination is recommended for a period of 1-6 months in children with adenotonsillar hypertrophy and with mild-to-moderate OSA [72], particularly and in children younger than 7 years and non-obese [73].

## 5. Weight Loss

The prevalence of OSA is higher in overweight or obese children [4]. The factors involved are different and include increased prevalence of adenotonsillar hypertrophy among obese children, altered neuromuscular tone during sleep resulting in higher upper airway collapsibility, central adiposity, and excess mechanical load on the chest wall resulting in its reduced excursion [74]. Moreover, OSA persists in about 50% of obese children after surgery [75]. For all these reasons, weight loss is crucial in overweight or obese children to reduce pharyngeal collapse or dysfunction [76,77]. Weight loss can improve breathing patterns as well as the quality of sleep and reduce daytime sleepiness [78].

Xanthopoulos et al. evaluated the effect of weight loss on OSA in 61 obese teenagers (mean age 14.8 ± 2.3 years) and AHI ≥ 2 [79]. They demonstrated how weight loss was successful in reducing symptoms with a positive association between the severity of OSA at the start of the treatment, the amount of weight loss achieved, and a concomitant reduction of symptoms. When less invasive traditional approaches have failed, bariatric surgery may represent an option for a selected group of extremely obese adolescents [80,81]. Further studies are needed to evaluate the short- and long-term efficacy of weight loss on OSA in children.

## 6. Conclusions

The first-line approach in pediatric populations with OSA is summarized in Table 1.

**Figure 1:**
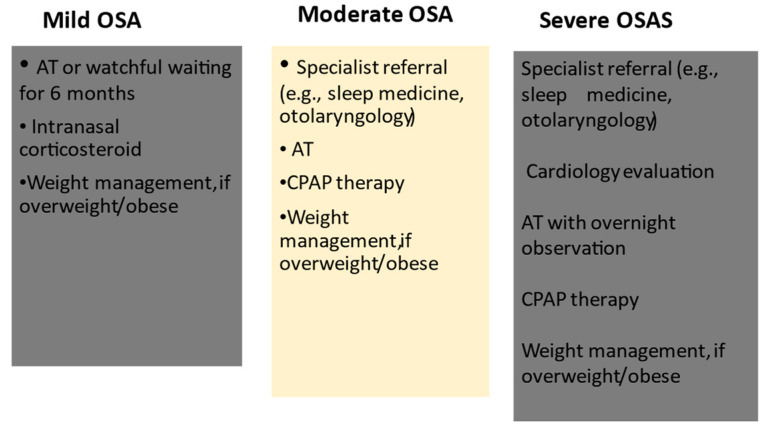
Algorithm for OSA management depending on pediatric age according to disease’s severity. AT, adenotonsillectomy; CPAP, continuous Positive Airway Pressure.

Figure 1 summarizes the algorithm for OSA management depending on pediatric age and according to the disease’s severity.

The main limitation of this review is its narrative nature because there are very few randomized studies on OSA management in children and adolescents. It is not possible to realize a systematic review, therefore, we performed an extensive literature analysis. Evaluation of the manuscripts published on this topic in the last 20 years showed that AT is the first-line treatment in most patients with adenotonsillar hypertrophy but medical therapy in children over 2 years old with mild OSA is a valid alternative.

In mild OSA, a 1- to 6-month trial with INS alone or in combination with montelukast can be considered. The clinical–instrumental monitoring of patients on medical therapy is crucial to assess the efficacy and consider other options. To monitor the efficacy of medical therapy, polysomnography should be repeated after 12 weeks. If polysomnography is not available, polygraphy, nocturnal oximetry, or capnography should be used to monitor treatment results. Further studies are needed to develop an algorithm that permits the selection of children with OSA who would benefit from alternatives to surgery, to identify those who could have better outcomes with an adenoidectomy or AT, to define the optimal bridge therapy before surgery, to evaluate the long-term effects of INS +/− LTRA, and to compare the impact of standardized approaches for weight loss.

## Figures and Tables

**Table 1:** Indications for medical therapy in pediatric populations with OSA.

Medical Therapy in OSA			
When?	Who?	What?	Why?
- Mild and uncomplicated OSA - When surgery cannot be performed	In children >2 years with adenotonsillar hypertrophy	- Intranasal corticosteroids (INS) - Antileukotriens (LTRA) - INS and LTRA - Weight Loss	- To decrease inflammation and infection in lymphoid tissues; also to reduce pharyngeal collapse
- As bridge therapy before surgery in severe OSA		- Systemic corticosteroids	- As rescue therapy
- During CPAP	In all patients with OSA requiring ventilation therapy	- Intranasal corticosteroids	- To reduce nasal congestion and rhinorrhea
- Residual forms after surgery	Children and adolescents undergoing surgery for craniofacial malformation or AT.	- Intranasal corticosteroids - LTRA - INS and LTRA - Weight Loss	- To resolve persistent symptoms such as nasal obstruction - To reduce pharyngeal collapse

CPAP: continuous positive airway pressure; AT, adenotonsillectomy.

## Data Availability

Not applicable.
